# BRAF inhibitor treatment of classical hairy cell leukemia allows successful vaccination against SARS-CoV-2

**DOI:** 10.1007/s00277-022-05026-z

**Published:** 2022-12-10

**Authors:** Judith Konrat, Wiebke Rösler, Michael Roiss, Fabienne Meier-Abt, Corinne C. Widmer, Stefan Balabanov, Markus G. Manz, Thorsten Zenz

**Affiliations:** 1 Department of Medical Oncology and Hematology, University Hospital Zürich and University of Zürich, Raemistrasse 100, CH-8091 Zürich, Switzerland; 2 Institute of Medical Genetics, University of Zürich, Zürich, Switzerland; 3 Department of Hematology, University Hospital of Basel, Basel, Switzerland

**Keywords:** Classical hairy cell leukemia, Vemurafenib, SARS-CoV-2 vaccination

## Abstract

In classical hairy cell leukemia (HCL), standard treatments including purine analogs achieve a durable response (up to 90%), but lead to severe immunosuppression and long-lasting depletion of CD4 + T lymphocytes. The BRAF inhibitor vemurafenib is effective in HCL, but its use in first-line treatment is restricted to select clinical situations (e.g. active infection). Its impact on immune function or response to vaccines in HCL is unclear. We treated four HCL patients with vemurafenib during the COVID-19 pandemic and monitored immune reconstitution and response to SARS-CoV-2 immunization. All patients responded to HCL treatment with normalization of peripheral blood counts. No severe infections occurred. As an indication of limited immunosuppression by vemurafenib, stable CD4 + and CD8 + T lymphocyte counts and immunoglobulin levels were observed. Three out of four patients received SARS-CoV-2 vaccination (Pfizer-BioNTech) during treatment with vemurafenib. IgG antibody levels against the spike-protein of SARS-CoV-2 were detected (40–818 AE/ml). Our data suggest that vemurafenib has limited effects on cellular and humoral immune function in HCL, which allows for successful SARS-CoV-2 vaccination. These data support the use of BRAF inhibitors during the current pandemic where continued immune response is necessary for minimizing the COVID-19-related risk of non-vaccinated patients.

## Introduction

Classical hairy cell leukemia (HCL) is a rare B-cell malignancy associated with intrinsic immunosuppression often complicated by pancytopenia or infection. The use of standard treatment options achieves a durable response, and patients have a life expectancy close to the normal population. The 2017 consensus guidelines for the diagnosis and management of patients with classical hairy cell leukemia recommend initial therapy with purine nucleoside analogs such as cladribine and pentostatin that are associated with prolonged and profound immunosuppression [1, 2]. Hence the additional treatment-induced immune deficiency is putting these patients at a high risk for infections. In view of the current coronavirus disease 2019 (COVID-19) pandemic, this is particularly relevant as patients with hematological cancers are more vulnerable to severe acute respiratory syndrome coronavirus 2 (SARS-CoV-2) and have a higher case fatality rate [3, 4].

Anti-CD20 antibody treatment with rituximab is known to hinder effective influenza vaccination at least 6 months after the last dose. Data for HCL patients are very limited. A study of the response to SARS-CoV-2 vaccination of patients with different hematologic malignancies also included seven HCL patients. All of these patients showed detectable levels of antibodies, but little is known about the disease or treatment status [5]. In patients with CLL exposed to anti-CD20 antibody treatment, there was no serologic response to SARS-CoV-2 vaccination within 12 months after the last treatment [6]. Patients who completed anti-CD20 antibody treatment at least 12 months before SARS-CoV-2 vaccination were more likely to show a serologic response (45.5%) [6]. B-cell lymphoma patients on treatment or within 9 months after treatment only rarely showed a significant IgG response after SARS-CoV-2 vaccination (11%), whereas 88% of the patients responded when vaccinated more than 9 months after the last treatment [4].

The vast majority of HCL patients show BRAF V600E mutations, causing activation of the MEK-ERK pathway and leading to enhanced cell proliferation, survival, and, ultimately, neoplastic transformation [1, 7]. The orally administered BRAF inhibitor (BRAFi) vemurafenib is efficient as monotherapy with an overall response rate of 96–100%, a complete response rate of 35–42%, and a median relapse-free survival of 19 months [8–10]. Currently, its use as monotherapy is limited to select clinical situations such as active infection or later-stage disease. In combination with rituximab, vemurafenib is associated with a durable response in most HCL patients [11].

While vemurafenib treatment may not be expected to impair immune function in HCL patients, surprisingly, little is known how BRAFi treatment may affect normal immune function and response to vaccination. Data from patients with melanoma treated with vemurafenib suggest limited toxic effects on the viability or function of lymphocytes [12, 13].

## Methods

In the period from October 2020 to August 2021, we have treated four HCL patients with vemurafenib after obtaining consent from patients and coverage from the respective insurance companies. All four patients consented the use of their medical data for research purposes. The main characteristics of the patients are reported in Table 1. All patients had an indication for treatment due to cytopenia. We included one treatment naïve patient. During treatment, immune status was monitored by measuring leukocyte subpopulations (lymphocytes, CD19 + B-lymphocytes, CD 4 + /8 + T lymphocytes, monocytes, neutrophil granulocytes, natural killer cells) and immunoglobulins (IgA, IgG, IgM) at least once a month. Response to BRAFi treatment was assessed by measuring hematologic parameters, immunophenotypic analysis by flow cytometry in peripheral blood, and detection of BRAF *V600E* allele burden with droplet digital PCR of genomic DNA. We have followed the treatment approach as previously described and started with an initial dosage of vemurafenib 2 × 240 mg/day [1, 14]. Upon residual disease detected by flow cytometry of the peripheral blood after 3–4 months (despite normal peripheral blood counts), a dose escalation of vemurafenib to 2 × 480 mg/day was performed. Anti-CD20 antibody treatment with rituximab (4 cycles of rituximab at a dosage of 375 mg/m2 every 2 weeks) was administered after successful SARS-CoV2 vaccination [11].

**Table 1:**
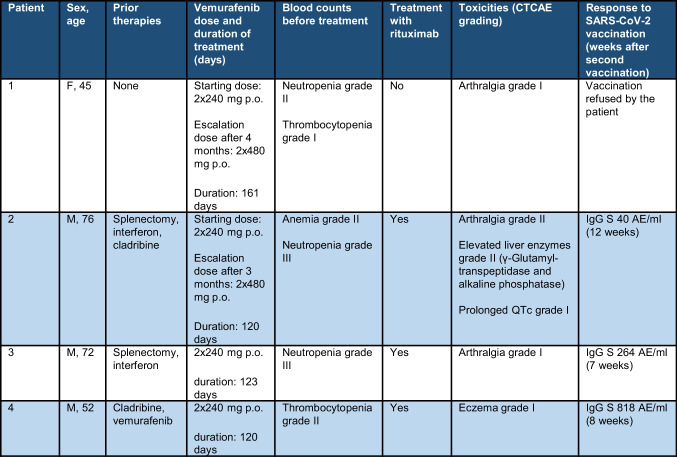
Patient characteristics

## Results and discussion

**Fig. 1:**
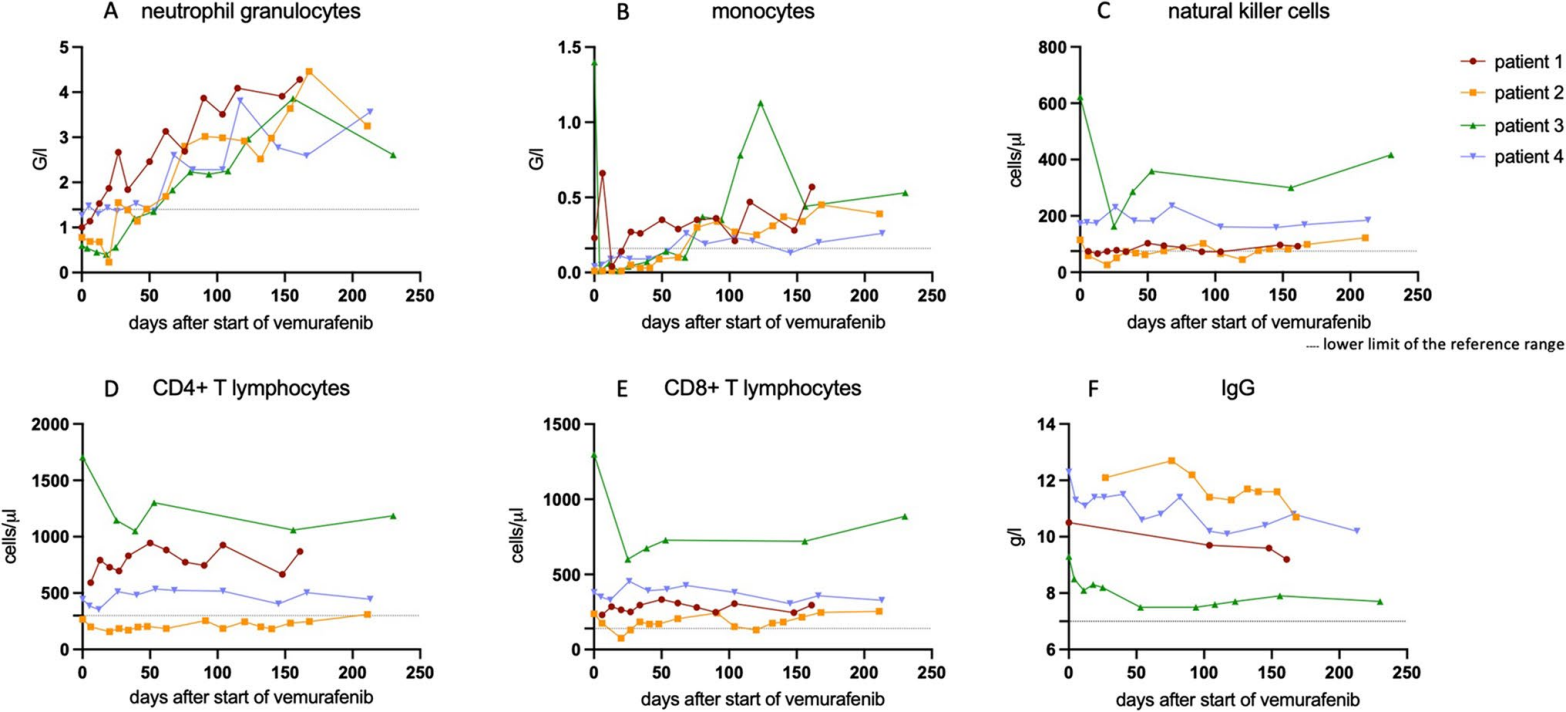
Neutrophil granulocytes, monocytes, natural killer cells, CD4 + T lymphocytes, CD8 + T lymphocytes and IgG levels during treatment interval (**A**–**F**)

All patients responded to therapy with vemurafenib (2 × 240 mg/day) with normalization of the peripheral blood counts (Fig. 1A, B). In patients 2 and 3, aggravation of neutropenia was observed in the first 3 weeks before complete hematological regeneration. The mean time to normalization of the peripheral blood counts was 65 days (range 13–132 days). Normalization of sIL-2 levels was observed in all patients after an average of 52.5 days (range 19–132 days).

None of the patients reached complete remission and flow cytometry of the peripheral blood showed hairy cells (range 0.03–3.6%). The BRAF *V600E* allele was detectable in all patients (best response P1: from 10.3 to 1.15%; P2: from 7.11 to 0.08%; P3: 0.69% (not measured before initiation of treatment); P4: 4% (not measured before initiation of treatment)).

During follow-up, no severe infections occurred. All patients had stable CD4 + T lymphocyte levels (mean value for each patient between 205 cells/μl and 1301 cells/μl) (Fig. 1C). Patient 2 was pretreated with cladribine and had low CD4 + T lymphocyte counts before treatment started. Stable CD8 + T lymphocyte levels (mean value for each patient between 170 and 831 cells/μl) were observed (Fig. 1D). No significant reduction was noted for natural killer cells (mean value for each patient between 75 and 358 cells/μl) (Fig. 1E). Immunoglobulin levels were normal in all patients without decline (range 7.5–12.7 g/l) (Fig. 1F).

Patient 1 refused the SARS-CoV2 vaccination, and therefore, no anti-CD20 antibody treatment was given. A dose escalation of vemurafenib to 2 × 480 mg/day was done. The residual disease still could be detected after 161 days of total treatment duration, and the therapy with vemurafenib was stopped.

All other patients received the SARS-CoV-2 vaccination (Pfizer-BioNTech) during vemurafenib treatment (first dose—P2 51 days, P3 32 days, and P4 39 days after initiation), which allowed us to study the antibody response of HCL patients on vemurafenib. The IgG antibody levels against the spike-protein of SARS-CoV-2 were determined in all patients. We found positive IgG S 40–818 AE/ml 7–12 weeks after the second vaccination (Table 1). IgG antibodies against the nucleocapsid protein of SARS-CoV-2 were undetectable, suggesting no infection with SARS-CoV-2 before the vaccination. In all three successfully vaccinated patients, rituximab was given 3 months after vemurafenib start to deepen the response.

In HCL patients, purine analogs or anti-CD20 antibody treatment lead to the depletion of T and/or B cells. Anti-CD20 antibody treatment is known to be associated with impaired response to vaccination, and it is recommended to vaccinate only 6 months after the last dose [14, 15]. Our findings suggest that the BRAFi vemurafenib has limited effect on cellular and humoral immune function. These data support prior results in melanoma, where no immune-related toxicity was shown [12, 13]. All of the patients who received the SARS-CoV-2 vaccination showed an immune response to the vaccine during the treatment with vemurafenib. Our data provide evidence for the role of BRAF inhibitor treatment for patients who require a continued immune response in order to e.g. successfully deliver a vaccination. This is particularly important in the recent COVID-19 pandemic and diseases such as HCL.

## References

[1] Grever MR, Abdel-Wahab O, Andritsos LA, Banerji V, Barrientos J, Blachly JS, Call TG, Catovsky D, Dearden C, Demeter J, Else M, Forconi F, Gozzetti A, Ho AD, Johnston JB, Jones J, Juliusson G, Kraut E, Kreitman RJ, Larratt L, Lauria F, Lozanski G, Montserrat E, Parikh SA, Park JH, Polliack A, Quest GR, Rai KR, Ravandi F, Robak T, Saven A, Seymour JF, Tadmor T, Tallman MS, Tam C, Tiacci E, Troussard X, Zent CS, Zenz T, Zinzani PL, Falini B (2017). Consensus guidelines for the diagnosis and management of patients with classic hairy cell leukemia. Blood.

[2] Cross M, Dearden C (2020). Hairy cell Leukaemia. Curr Oncol Rep.

[3] He W, Chen L, Chen L, Yuan G, Fang Y, Chen W, Wu D, Liang B, Lu X, Ma Y, Li L, Wang H, Chen Z, Li Q, Gale RP (2020). COVID-19 in persons with haematological cancers. Leukemia.

[4] Ghione P, Gu JJ, Attwood K, Torka P, Goel S, Sundaram S, Mavis C, Johnson M, Thomas R, McWhite K, Darrall A, DeMarco J, Kostrewa J, Mohr A, Rivas L, Neiders M, Suresh L, Segal BH, Griffiths EA, Ramsperger V, Shen L, Hernandez-Ilizaliturri FJ (2021). Impaired humoral responses to COVID-19 vaccination in patients with lymphoma receiving B-cell-directed therapies. Blood.

[5] Greenberger LM, Saltzman LA, Senefeld JW, Johnson PW, DeGennaro LJ, Nichols GL (2021). Antibody response to SARS-CoV-2 vaccines in patients with hematologic malignancies. Cancer Cell.

[6] Herishanu Y, Avivi I, Aharon A (2021). Efficacy of the BNT162b2 mRNA COVID-19 vaccine in patients with chronic lymphocytic leukemia. Blood.

[7] Tiacci E, Trifonov V, Schiavoni G (2011). BRAF mutations in hairy-cell leukemia. N Engl J Med.

[8] Dietrich S, Pircher A, Endris V, Peyrade F, Wendtner CM, Follows GA, Hüllein J, Jethwa A, Ellert E, Walther T, Liu X, Dyer MJ, Elter T, Brummer T, Zeiser R, Hermann M, Herold M, Weichert W, Dearden C, Haferlach T, Seiffert M, Hallek M, von Kalle C, Ho AD, Gaehler A, Andrulis M, Steurer M, Zenz T (2016). BRAF inhibition in hairy cell leukemia with low-dose vemurafenib. Blood.

[9] Dietrich S, Glimm H, Andrulis M, von Kalle C, Ho AD, Zenz T (2012). BRAF inhibition in refractory hairy-cell leukemia. N Engl J Med.

[10] Tiacci E, Park JH, De Carolis L, Chung SS, Broccoli A, Scott S, Zaja F, Devlin S, Pulsoni A, Chung YR, Cimminiello M, Kim E, Rossi D, Stone RM, Motta G, Saven A, Varettoni M, Altman JK, Anastasia A, Grever MR, Ambrosetti A, Rai KR, Fraticelli V, Lacouture ME, Carella AM, Levine RL, Leoni P, Rambaldi A, Falzetti F, Ascani S, Capponi M, Martelli MP, Park CY, Pileri SA, Rosen N, Foà R, Berger MF, Zinzani PL, Abdel-Wahab O, Falini B, Tallman MS (2015). Targeting mutant BRAF in relapsed or refractory hairy-cell leukemia. N Engl J Med.

[11] Tiacci E, De Carolis L, Simonetti E, Capponi M, Ambrosetti A, Lucia E, Antolino A, Pulsoni A, Ferrari S, Zinzani PL, Ascani S, Perriello VM, Rigacci L, Gaidano G, Della Seta R, Frattarelli N, Falcucci P, Foà R, Visani G, Zaja F, Falini B (2021). Vemurafenib plus rituximab in refractory or relapsed hairy-cell leukemia. N Engl J Med.

[12] Ilieva KM, Correa I, Josephs DH, Karagiannis P, Egbuniwe IU, Cafferkey MJ, Spicer JF, Harries M, Nestle FO, Lacy KE, Karagiannis SN (2014). Effects of BRAF mutations and BRAF inhibition on immune responses to melanoma. Mol Cancer Ther.

[13] Comin-Anduix B, Chodon T, Sazegar H, Matsunaga D, Mock S, Jalil J, Escuin-Ordinas H, Chmielowski B, Koya RC, Ribas A (2010). The oncogenic BRAF kinase inhibitor PLX4032/RG7204 does not affect the viability or function of human lymphocytes across a wide range of concentrations. Clin Cancer Res.

[14] Grever M, Andritsos L, Banerji V, Barrientos JC, Bhat S, Blachly JS, Call T, Cross M, Dearden C, Demeter J, Dietrich S, Falini B, Forconi F, Gladstone DE, Gozzetti A, Iyengar S, Johnston JB, Juliusson G, Kraut E, Kreitman RJ, Lauria F, Lozanski G, Parikh SA, Park J, Polliack A, Ravandi F, Robak T, Rogers KA, Saven A, Seymour JF, Tadmor T, Tallman MS, Tam CS, Tiacci E, Troussard X, Zent C, Zenz T, Zinzani PL, Wörmann B. (2021) Hairy cell leukemia and COVID-19 adaptation of treatment guidelines. Leukemia. 35(7):1864–1872 10.1038/s41375-021-01257-7 PMC8093591 33947938

[15] Yri OE, Torfoss D, Hungnes O (2011). Rituximab blocks protective serologic response to influenza A (H1N1) 2009 vaccination in lymphoma patients during or within 6 months after treatment. Blood.

